# Case Report: Treatment Planning Study to Demonstrate Feasibility of Transthoracic Ultrasound Guidance to Facilitate Ventricular Tachycardia Ablation With Protons

**DOI:** 10.3389/fcvm.2022.849247

**Published:** 2022-05-04

**Authors:** Rosalind Perrin, Patrick Maguire, Adriano Garonna, Georg Weidlich, Shelley Bulling, Marie Fargier-Voiron, Cedric De Marco, Eleonora Rossi, Mario Ciocca, Viviana Vitolo, Alfredo Mirandola

**Affiliations:** ^1^EBAMed SA, Geneva, Switzerland; ^2^MedDevicePharma LLC, Foster City, CA, United States; ^3^Radiation Oncology, National Medical Physics and Dosimetry Company, Palo Alto, CA, United States; ^4^Clinique de Genolier, Genolier, Switzerland; ^5^Centro Nazionale di Adroterapia Oncologica (CNAO), Pavia, Italy

**Keywords:** ultrasound, ventricular tachycardia, protons, stereotactic radioablation, cardiac motion monitoring

## Abstract

**Background:**

Cardiac arrhythmias, such as ventricular tachycardia, are disruptions in the normal cardiac function that originate from problems in the electrical conduction of signals inside the heart. Recently, a non-invasive treatment option based on external photon or proton beam irradiation has been used to ablate the arrhythmogenic structures. Especially in proton therapy, based on its steep dose gradient, it is crucial to monitor the motion of the heart in order to ensure that the radiation dose is delivered to the correct location. Transthoracic ultrasound imaging has the potential to provide guidance during this treatment delivery. However, it has to be noted that the presence of an ultrasound probe on the chest of the patient introduces constraints on usable beam angles for both protons and photon treatments. This case report investigates the possibility to generate a clinically acceptable proton treatment plan while the ultrasound probe is present on the chest of the patient.

**Case:**

A treatment plan study was performed based on a 4D cardiac-gated computed tomography scan of a 55 year-old male patient suffering from refractory ventricular tachycardia who underwent cardiac radioablation. A proton therapy treatment plan was generated for the actual treatment target in presence of an ultrasound probe on the chest of this patient. The clinical acceptability of the generated plan was confirmed by evaluating standard target dose-volume metrics, dose to organs-at-risk and target dose conformity and homogeneity.

**Conclusion:**

The generation of a clinically acceptable proton therapy treatment plan for cardiac radioablation of ventricular tachycardia could be performed in the presence of an ultrasound probe on the chest of the patient. These results establish a basis and justification for continued research and product development for ultrasound-guided cardiac radioablation.

## Introduction

Treatment of cardiac arrhythmias using a non-invasive treatment technique based on external beam radiation has recently shown promising results (1–6). This technique involves delivery of photon or proton beams in a single out-patient session with the aim to stop the electrical conduction in the arrhythmogenic substrate. The surrounding tissues, typically referred to as organs-at-risk (OARs), should be spared from radiotoxic effects as much as possible. This might be achieved, for example, by choosing protons beams over photons beams, as proton therapy has to ability to precisely deliver a radiation dose via the Bragg peak phenomenon (7).

In addition to beam choice, it is of critical importance to take cardiac motion into account during treatment planning and treatment beam delivery. Several solutions have been proposed to handle the cardiac motion during treatment including enlargement of the treatment targets with margins (3, 8) or inferring the cardiac motion based on ECG signals (9–11), electrical impedance signals or X-ray imaging of implanted leads (3, 12). The limitations of these solutions are, among others, the requirement to implant fiducial markers, additional ionizing radiation dose deposition to the patient and the need for a motion surrogate (13).

Transthoracic ultrasound (US) imaging allows for real-time cardiac motion monitoring during the treatment. This image modality has been used for radiation therapy guidance for oncological targets before (14–16) and it overcomes some limitations associated with the currently available motion monitoring solutions. The usage of US imaging, however, requires placing an US probe on the chest of the patient. The presence of the US probe in the path of the radiation beam during the treatment can potentially cause dose delivery errors, which may influence the treatment outcome of the patient.

In literature several options to deal with the presence of an US probe during photon radiation treatment of oncological targets have been described (12, 17–19). To the best of our knowledge, none of the published works focused on dealing with a US probe during the irradiation of cardiac targets with protons. For this reason, this work presents a case report of a patient with ventricular tachycardia (VT) for whom a proton treatment planning study was performed. The aim of this treatment planning study was to design a clinically acceptable cardiac radioablation proton treatment plan for a real VT target.

## Case Description

For this proof-of-concept study the 4D cardiac-gated CT scan from a 55 year-old male patient suffering from VT was used. The CT data of this VT patient has been previously used for other purposes in a work published by Gianni et al. (20). The treatment target for this patient had a size of 45 cm^3^ and it was located on the left ventricular free wall. This clinical target volume (CTV) was determined by electrophysiological mapping and contoured prior to the treatment by a medical doctor from the Texas Heart Arrhythmia Institute in Austin, USA. The left anterior descending coronary artery, the circumflex coronary arteries and the non-involved left ventricle were OARs near the target.

**Figure 1:**
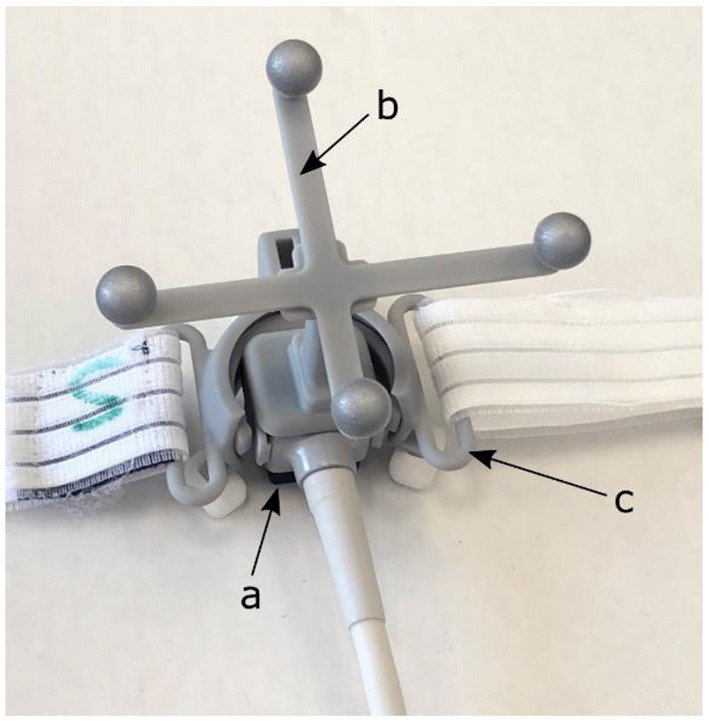
Prototype version of the EBAMed proprietary US probe system (a) equipped with an optical localization marker (b) and a probe holder with strap (c) that allows for fixation to the patient's chest.

First, the 4D CT scan of the VT patient was loaded into the Raysearch^®^ Raystation treatment planning system (version 10B, Raysearch Laboratories AB, Stockholm, Sweden). Subsequently, a virtual representation of the prototype version of the proprietary US probe system of EBAMed (Geneva, Switzerland) was manually inserted as volume of interest (VOI) in two locations representing the estimated position of the apical and parasternal US viewing windows. A separate study has already verified that these US viewing windows provide US images of sufficient quality for VT patients in supine position (21). The US probe was simulated as a cube of 2 × 2 × 2 cm. It is equipped with infra-red markers such that the probe can be localized by an optical camera (see Figure 1) and it is attached to a holder such that it can be fixed on the chest of the patient allowing for hands-free imaging during the treatment. To account for uncertainties in repositioning of the US probe during the treatment, including probe position uncertainties due to respiration and breath-hold differences, an isotropic safety margin of 10 mm has been added to the union of the US probe, holder, and optical marker.

The parasternal US probe position allowed entrance of the treatment beams from optimal directions with respect to dosimetry for this particular patient. After selection of this virtual US probe position, a pencil-beam scanned proton therapy treatment plan was generated with the treatment planning system using the CNAO (Pavia, Italy) synchrotron proton beam model adapted to the Hitachi PROBEAT gantry system with 360° range of beam angles (22). During planning, the solid angle was restricted to take into account the US probe, the probe holder and the localization marker. Two fields were applied both with a gantry angle of 25° and a couch rotation of 0° and 90° for beam 1 and 2, respectively. The treatment volume was planned with an internal target volume (ITV) approach in order to compensate for shape and position changes of the target due to the heartbeat. It was assumed that the motion of the heart due to respiration would be mitigated using a breath-hold technique or respiratory gating. The envisioned role of the US imaging during this treatment was real-time cardiac motion monitoring and sending an alert to the operator in case the measured motion was outside of predefined limits.

For the generation of the ITV, the heartbeat motion envelope was extracted from the 4D CT scan by deformable registration of each phase of the 4D CT scan to the planning CT scan. The resulting ITV is the union of the CTVs at all phases of the 4D CT. Finally, the planning target volume (PTV) was generated by adding a 5 mm margin to the ITV based on typical patient set-up errors which are expected when no image guidance tool like US imaging is used.

Dose constraints on dose-volume tolerances (Table 1) in agreement with prior investigators were set as planning objectives. All doses are reported in Cobalt Gray Equivalent Dose (CGyE). The plan required the ITV to be covered by the 25 CGyE isodose, which is a dose level used in prior clinical studies to achieve safe, efficacious radioablation. To achieve this, the plan was normalized so that PTV D92% = 25 CGyE. Also, in order to arrive at a satisfactory treatment plan (26, 27), robust optimization with 2 mm set-up error in all directions and 2% range uncertainty was used during planning.

**Table 1:** Evaluation metrics for a clinically acceptable plan (all constraints must be satisfied for a plan to be considered clinically acceptable).

**Structure**	**Dose-volume metric**	**Dose-volume limit**	**Source dose-volume limit**
Target volume	D95%	100% dose (25.0 CGyE) to 95% volume	Prescription isodose (100%)
Target volume	D2% (near max dose)	120% dose (30 CGyE) to 2% volume	Hot spot allowable in target volume up to 120% of prescription dose for stereotactic body RT (23)
Target volume	D98% (near min dose)	95% dose (23.75 CGyE) to 98% volume	Cold spot allowable at 95% prescription isodose
Spinal cord	D (max)	7 CGyE	(23)
Coronary arteries	D (max)	14 CGyE	(24)
Skin	V (23Gy)	10 cm^3^	(23)
ICD	D (0.03cc)	2 CGyE	(25)
Aorta	D (max)	20 CGyE	(24)

To verify the clinical acceptability of the generated plan, evaluation of standard target dose-volume metrics D98, D95 D50 and D2 was performed. In addition, the dose to OARs and the target dose conformity and homogeneity were evaluated.

## Discussion

**Figure 2:**
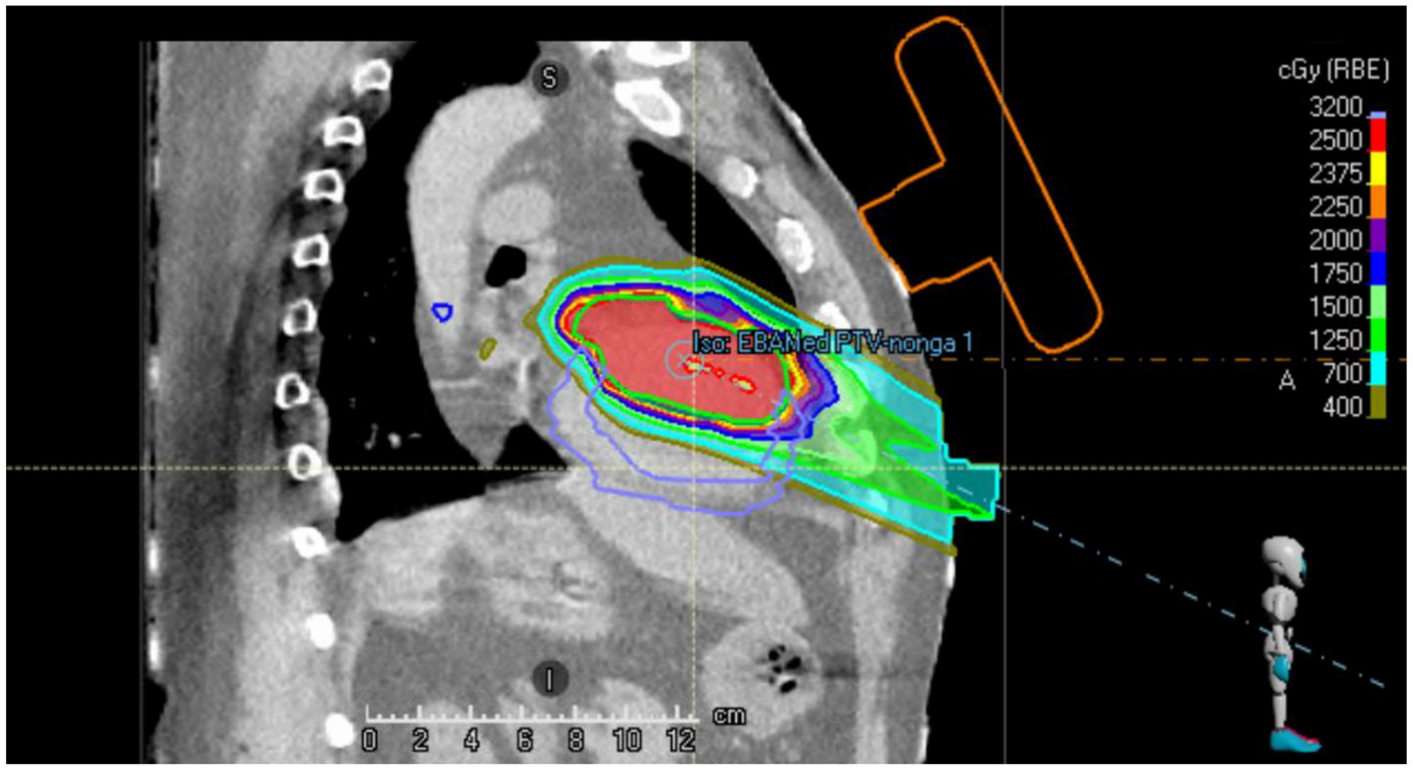
Sagittal slice of the single beam proton plan generated for the VT patient. The location of the virtual US probe with localization marker on the chest of the patient is shown in orange.

Figure 2 shows a sagittal slice of the proton treatment plan that has been generated for the patient studied in this case report. It can be observed that the beams do not intersect the orange contour of the virtual US probe.

Table 2 details the proton treatment plan characteristics. Target coverage and dose conformity as well as sparing of OARs, were found to be acceptable. The D98 was less than the value required in Table 1, due to the coronary arteries abutting the PTV in the superior extent of the target. Limiting the dose received by these structures was prioritized over target coverage in this region of the target.

**Table 2:** Proton treatment plan characteristics.

**Dosimetric parameter**	**Value**
ITV -> PTV margin	5 mm
D95 (ITV)	25.1 CGyE
D98 (ITV)	21.8 CGyE
D2 (ITV)	30.2 CGyE
D50 (ITV)	26.6 CGyE
Homogeneity Index (ITV)	0.32
Conformity Index to PTV	1.02
Minimum beam energy	81.0 MeV
Maximum beam energy	160.5 MeV
Dose to Nearby OARs	
• Non-involved left ventricle (V20Gy)	9.82 cm^3^
• Non-involved left ventricle (Dmean)	4.53 CGyE
• Left anterior descending coronary artery (D0.03cc)	10.7 CGyE
• Circumflex coronary arteries (D0.03cc)	9.42 CGyE

This case report describing a treatment planning study for a VT patient has shown that the use of an US probe in parasternal viewing position during treatment delivery will not prevent a clinically acceptable treatment with proton radiation for this particular patient. These findings establish a basis and justification for the continued research and product development to arrive at an integrated solution for ultrasound-guided cardiac radioablation. The usage of US imaging during the treatment will potentially allow for ITV margin reductions. However, before final conclusions can be drawn, more extensive treatment planning studies are necessary in which actual US probe positions (both parasternal and apical US viewing windows) instead of estimated probe positions are considered. In addition, future research efforts are planned to focus on improved OAR sparing, which can be achieved by more precise targeting. This can, for example, be accomplished by cardiac phase gating with a careful definition of the gate range, instead of only monitoring the cardiac motion as considered in this work.

## Data Availability Statement

The raw data supporting the conclusions of this article will be made available by the authors, without undue reservation.

## Ethics Statement

Ethical review and approval was not required for the study on human participants in accordance with the local legislation and institutional requirements. The patients/participants provided their written informed consent to participate in this study.

## Author Contributions

RP, AG, and MF-V design of work. RP and PM: data collection and drafting the article. RP, AM, ER, CD, VV, and PM: data analysis and interpretation. GW, PM, and SB: critical revision of article. AG, RP, PM, and MC: final approval of version to be published. All authors contributed to the article and approved the submitted version.

## Funding

This publication is part of a project that has received funding from the European Union's Horizon 2020 research and innovation program under grant agreement no. 954783.

## Conflict of Interest

PM is founder and owner of MedDevicePharma LLC. GW is founder and owner of National Medical Physics and Dosimetry Company. PM and GW are consultants to EBAMed SA. RP was previously employed and AG is still employed by EBAMed SA. The remaining authors declare that the research was conducted in the absence of any commercial or financial relationships that could be construed as a potential conflict of interest.

## Publisher's Note

All claims expressed in this article are solely those of the authors and do not necessarily represent those of their affiliated organizations, or those of the publisher, the editors and the reviewers. Any product that may be evaluated in this article, or claim that may be made by its manufacturer, is not guaranteed or endorsed by the publisher.
